# Arlequin (version 3.0): An integrated software package for population genetics data analysis

**Published:** 2007-02-23

**Authors:** Laurent Excoffier, Guillaume Laval, Stefan Schneider

**Affiliations:** Computational and Molecular Population Genetics Lab, Zoological Institute, University of Berne, Baltzerstrasse 6, 3012 Berne, Switzerland

**Keywords:** Computer package, population genetics, genetic data analysis, AMOVA, EM algorithm, gametic phase estimation, spatial expansion

## Abstract

Arlequin ver 3.0 is a software package integrating several basic and advanced methods for population genetics data analysis, like the computation of standard genetic diversity indices, the estimation of allele and haplotype frequencies, tests of departure from linkage equilibrium, departure from selective neutrality and demographic equilibrium, estimation or parameters from past population expansions, and thorough analyses of population subdivision under the AMOVA framework. Arlequin 3 introduces a completely new graphical interface written in C++, a more robust semantic analysis of input files, and two new methods: a Bayesian estimation of gametic phase from multi-locus genotypes, and an estimation of the parameters of an instantaneous spatial expansion from DNA sequence polymorphism. Arlequin can handle several data types like DNA sequences, microsatellite data, or standard multi-locus genotypes. A Windows version of the software is freely available on http://cmpg.unibe.ch/software/arlequin3.

## Introduction

Most genetic studies on non-model organisms require a description of the pattern of diversity within and between populations, based on a variety of markers often including mitochondrial DNA (mtDNA) sequences and microsatellites. The genetic data are processed to extract information on the mating system, the extent of population subdivision, the past demography of the population, or on departure from selective neutrality at some loci. A series of computer packages have been developed in the last 10 years to assist researchers in performing basic population genetics analyses like Arlequin2 (Schneider et al. 2000), DNASP (Rozas et al. 2003), FSTAT (Goudet 1995), GENEPOP (Raymond and Rousset 1995b), or GENETIX (Belkhir et al. 2004). These programs have been widely used in the molecular ecology and conservation genetics community (Labate 2000; Luikart and England 1999; Schnabel et al. 1998). Among these, Arlequin is a very versatile (though not universal) program, and complements the other programs listed above. It can handle several data types like RFLPs, DNA sequences, microsatellite data, allele frequencies, or standard multi-locus genotypes, while allowing the user to carry out the same types of analyses irrespective of the data types.

We present here the version 3 of Arlequin with additional methods extending its capacities for the handling of unphased multi-locus genotypes and for the estimation of parameters of a spatial expansion. Note that these new developments are mainly implementations of new methodologies developed in our lab. We believe these methods will be useful to the research community, but we do not claim that alternative methods implemented by other groups in other programs are inadequate. A new graphical interface has been developed to provide a better integration of the different analyses into a common framework, and an easier exploration of the data by performing a wide variety of analyses with different settings. The tight coupling of Arlequin with the simulation programs SIMCOAL2 (Laval and Excoffier 2004) and SPLATCHE (Currat et al. 2004) should also make it useful to describe patterns of genetic diversity under complex evolutionary scenarios.

## Methods implemented in Arlequin

Arlequin provides methods to analyse patterns of genetic diversity within and between population samples.

### Intra-population methods

- Computation of different standard genetic indices, like the number of segregating sites, the number of different alleles, the heterozygosity, the base composition of DNA sequences, gene diversity, or the population effective size *Ne* scaled by the mutation rate μ as θ = *4N_e_u*.
- Maximum-likelihood estimation of allele and haplotype frequencies via the EM algorithm (Excoffier and Slatkin 1995).
- Estimation of the gametic phase from multi-locus genotypes via the Excoffier-Laval-Balding (ELB) algorithm (Excoffier et al. 2003).
- Estimation of the parameters of a demographic (Rogers and Harpending 1992; Schneider and Excoffier 1999) or a spatial (Excoffier 2004; Ray et al. 2003) expansion, from the mismatch distribution computed on DNA sequences.
- Calculation of several measures of linkage disequilibrium (LD) like *D*, *D*’, or *r*^2^ (Hedrick 1987), and test of non-random association of alleles at different loci when the gametic phase is known (Weir 1996) or unknown (Slatkin and Excoffier 1996).
- Exact test of departure from Hardy-Weinberg equilibrium (Guo and Thompson 1992).
- Computation of Tajima’s *D* (Tajima 1989) and Fu’s F*_S_* (Fu 1997) statistics, and test of their significance by coalescent simulations (Hudson 1990; Nordborg 2003) under the infinite-site model.
- Tests of selective neutrality under the infinite-alleles model, like the Ewens-Watterson test (Slatkin 1996; Watterson 1978), and Chakraborty’s amalgamation test (Chakraborty 1990).

### Inter-population methods

- Search for shared haplotypes between populations
- Analysis of population subdivision under the AMOVA framework (Excoffier 2003; Excoffier et al. 1992), with three hierarchical levels: genes within individuals, individuals within demes, demes within groups of demes. Computation of *F*-statistics like the local inbreeding coefficient *F_IS_* or the index of population differentiation *F_ST_*.
- Computation of genetic distances between populations related to the pairwise *F_ST_* index (Gaggiotti and Excoffier 2000; Reynolds et al. 1983; Slatkin 1995).
- Exact test of population differentiation (Goudet et al. 1996; Raymond and Rousset 1995a).
- A simple assignment test of individual genotypes to populations according to their likelihood (Paetkau et al. 1997).
- Computation of correlations or partial correlations between a set of 2 or 3 distance matrices (Mantel test: Smouse et al. 1986)

## New features in Arlequin 3

- Version 3 of Arlequin integrates the core computational routines and the interface in a single program written in C++ for the Windows environment. The interface has been entirely redesigned to provide better usability.
- Incorporation of two new methods to estimate gametic phase and haplotype frequencies:
  ⋄ The ELB algorithm (Excoffier et al. 2003) is a pseudo-Bayesian approach aiming at reconstructing the gametic phase of multi-locus genotypes, and the estimation of the haplotype frequencies are a by-product of this process. Phase updates are made on the basis of a window of neighbouring loci, and the window size varies according to the local level of linkage disequilibrium.
  ⋄ The EM zipper algorithm, which is an extension of the EM algorithm for estimating haplotype frequencies (Excoffier and Slatkin 1995), aims at estimating the haplotype frequencies in unphased multi-locus genotypes. The estimation of the gametic phases are a by-product of this process. It proceeds by adding loci one at a time and progressively extending the length of the reconstructed haplo-types. With this method, Arlequin does not need to build all possible genotypes for each individual like in the conventional EM algorithm, but it only considers the genotypes whose sub-haplotypes have non-null estimated frequencies. It can thus handle a much larger number of polymorphic sites than the strict EM algorithm. It also gives final haplotype frequencies that often have a higher likelihood than those estimated under the strict EM algorithm, due to the difficulty in exploring the space of all possible genotypes when the number of polymorphic loci in the sample is large. Note that this version of the EM algorithm is equivalent to that implemented in the SNPHAP program by David Clayton fully described on http://www-gene.cimr.cam.ac.uk/clayton/software/snphap.txt, and whose efficiency for inferring gametic phase has been favorably evaluated (Adkins 2004).
- Estimation of the parameters of a spatial expansion (age of the expansion and deme size scaled by the mutation rate, as well as the number of migrants exchanged between neighbouring demes) from the patterns of polymorphism in a sample of DNA sequences. The estimation is based on a simple model of instantaneous and infinite range expansion, where some time ago, a single deme instantaneously colonized an infinite number of demes subsequently interconnected by migration (as under an infinite-island model) (Excoffier 2004). The parameters are obtained by a least-square approach maximizing the fit between the observed and expected distribution of pair-wise differences (the mismatch distribution) computed on DNA sequences. Confidence intervals of the estimates are obtained under a parametric bootstrap approach involving the simulation of an instantaneous expansion under a coalescent framework.
- Estimation of confidence intervals for *F*-statistics estimated under the AMOVA framework by bootstrapping over loci for multi-locus data. A minimum of 8 loci are necessary for the computation of these confidence intervals.
- A completely rewritten and more robust input file parsing procedure, giving more precise information on the location of potential syntax and format errors in input files.
- Use of the ELB algorithm described above to generate samples of phased multi-locus genotypes, which allows one to analyse unphased multi-locus genotype data as if the phase was known. The phased data sets are output in Arlequin projects that can be analysed in a batch mode to obtain the distribution of statistics taking phase uncertainty into account.
- New output files fully compatible with modern web browsers.

## Availability

A Windows executable version Arlequin ver 3 can be freely downloaded on http://cmpg.unibe.ch/software/arlequin3, together with an up-to-date user manual in Adobe Acrobat PDF format incorporating more technical details on the methods used in Arlequin 3, as well as several example files.
